# Thyroid nodules with highly suspicious ultrasonographic features, but with benign cytology on two occasions: is malignancy still possible?

**DOI:** 10.1590/2359-3997000000176

**Published:** 2016-08-01

**Authors:** Pedro Weslley Rosário, Maria Regina Calsolari

**Affiliations:** 1 Santa Casa de Belo Horizonte Belo Horizonte MG Brasil Santa Casa de Belo Horizonte, Belo Horizonte, MG, Brasil

**Keywords:** Thyroid nodule, benign cytology, ultrasonography

## Abstract

**Objective:**

There is no information about the frequency of malignancy specifically in the case of thyroid nodules with highly suspicious sonographic features, but with two fine needle aspiration (FNA) showing benign cytology. This was the objective of the study.

**Subjects and methods:**

We report the results of 105 patients with thyroid nodules considered “highly suspicious” according to the ultrasonographic classification of American Thyroid Association, in whom FNA revealed benign cytology on two occasions (interval of 6 months).

**Results:**

Thyroidectomy was performed in 11 cases due to desire of the patient or significant growth of the nodule. In these patients, cytology continued to be benign in 9, was non-diagnostic in 1, and suspicious in 1. Histology revealed papillary carcinoma in only one nodule. In patients in whom a third FNA was obtained for this study (n = 94), cytology continued to be benign in 86, became non-diagnostic in 5, indeterminate in 2, and suspicious in 1. The last 8 patients (with non-benign cytology) were submitted to thyroidectomy and histology revealed malignancy in only one nodule.

**Conclusion:**

The rate of malignancy found here for nodules with highly suspicious sonographic features, even after two FNA showing benign cytology, was 2%. We believe that in these cases, the continuation of follow-up consisting of ultrasound at intervals of 2 years may still be adequate.

## INTRODUCTION

Nodular thyroid disease is common in clinical practice and the most frequent result of fine-needle aspiration (FNA) is benign cytology. Despite the high negative predictive value of this result, a non-negligible percentage of nodules with benign cytology are carcinomas. Repetition of FNA is recommended in the case of nodules with initial benign cytology, but with “highly suspicious” findings on ultrasonography (US) (1-4). This recommendation is stated in the latest guideline of the American Thyroid Association (ATA) (4). Also according to this guideline, “If a nodule has undergone repeat US-guided FNA with a second benign cytology result, ultrasound surveillance for this nodule for continued risk of malignancy is no longer indicated” (4).

FNA was repeated in nodules with initial benign cytology in several studies. Some studies report the rate of malignancy for nodules with two or more FNA with benign cytology, but do not mention ultrasonographic features of the nodules; consequently, the frequency of malignancy specifically in the case of nodules with highly suspicious US appearance was not reported (5,6). Other studies report the result of a second FNA for nodules with highly suspicious US features and initial benign cytology, but additional investigation was only performed on few nodules when cytology continued to be benign (1-3,7). This question is pertinent since studies suggest that three (and not only two) FNA showing benign cytology are necessary for virtual exclusion of malignancy (5,6,8). To our knowledge, there is no information about the frequency of malignancy specifically in the case of nodules with highly suspicious US features, but with two FNA showing benign cytology, and this is necessary to recommend how these cases should be followed up.

## SUBJECTS AND METHODS

We report here the results of 105 patients [90 women and 15 men, age of 16 to 80 years (median 46 years), and TSH concentrations of 0.5 to 8 mIU/L (median 2 mIU/L)] with thyroid nodules [diameter of 6 to 40 mm (median 23 mm), ≥ 10 mm in 90/105] considered “highly suspicious” according to the ultrasonographic classification of ATA (4), in whom FNA revealed benign cytology on two occasions (interval of 6 months) (1,3). None of the patients had a family history of thyroid carcinoma, exposure to radiation during childhood or adolescence, hypercalcitoninemia, compressive symptoms, or a nodule detected incidentally by FDG-PET for the staging of oncological disease (1,3). None of the patients received laser- or radiofrequency ablation, radioiodine therapy, percutaneous ethanol injection, or TSH suppression.

US was performed with a linear multifrequency 12-14 MHz transducer for morphological analysis (B-mode) and for power Doppler evaluation. The images were analyzed by experienced professionals before FNA. Solid hypoechoic nodule or a solid hypoechoic component in a partially cystic nodule with one or more of the following features were defined as “highly suspicious”: irregular margins (specifically defined as infiltrative, microlobulated, or spiculated), microcalcifications, taller than wide shape, disrupted rim calcifications with small extrusive hypoechoic soft tissue component, or evidence of extrathyroidal extension (4).

After the second FNA had confirmed the benign nature of the nodule, all patients were followed up by the repetition of US at intervals of 6 months. During follow-up, thyroidectomy was indicated by desire of the patient or when the nodule exhibited significant growth and had a diameter ≥ 2 cm (both). Significant growth was defined as an increase > 50% in the initial volume of the nodule (1,3,4), calculated as length x width x depth x 0.52. FNA (third) was obtained before thyroidectomy. For this study, all patients who had not undergone surgery were submitted to a new FNA (third) and neck US specifically for the investigation of suspicious lymph nodes. Histology was obtained in cases in which the third FNA did not reveal benign cytology.

FNA was always guided by US and the samples were analyzed by pathologists experienced in thyroid cytology, who were unaware of the result of US and of the previous cytologies. The cytological diagnosis was classified as nondiagnostic, benign, indeterminate [follicular lesion or atypia of undetermined significance, suspicious for or follicular neoplasm], suspicious for malignancy, or malignant (4).

The study was approved by the local Research Ethics Committee.

## RESULTS

Thyroidectomy was performed in 11 cases due to desire of the patient (n = 2) or significant growth of the nodule (n = 9). In these patientes, cytology continued to be benign in 9 (82%), was non-diagnostic in 1 (9%), and suspicious in 1 (9%). Histology revealed papillary carcinoma in only one nodule (with significant growth and third FNA showing suspicious cytology) (tumor stage T1N1aM0).

In patients in whom a third FNA was obtained for this study (n = 94), cytology continued to be benign in 86 (91.5%), became non-diagnostic in 5 (5.3%), indeterminate in 2 (2.1%), and suspicious in 1 (1%). The last 8 patients (with non-benign cytology) were submitted to thyroidectomy and histology revealed malignancy in only one nodule (third FNA with suspicious cytology) (tumor stage T3NxM0).

Thus, the rate of malignancy found here for nodules with highly suspicious US features (4), even after two FNA showing benign cytology, was 2%. The two cases of malignancy were classical papillary carcinomas. None of the patients had suspicious lymph nodes on neck US.

## DISCUSSION

Although US findings and the result of FNA are usually concordant, approximately 10% of thyroid nodules with initial benign cytology are highly suspicious for malignancy on US (1-4). In this situation, repetition of FNA after a few months is currently recommended (1-4). In the remaining nodules with benign cytology, repetition of FNA should only be considered in the case of significant growth or appearance of suspicious US features (4). According to the recent ATA guidelines, US surveillance for this nodule for continued risk of malignancy is no longer indicated after two FNA with benign cytology (4). To define the best follow-up strategy in these cases, it is fundamental to know the frequency of malignancy of these nodules. Greater concern exists in the case of nodules with highly suspicious US features. Previous studies reported the result of a second FNA for nodules with highly suspicious US features and initial benign cytology, but additional investigation was only performed on few nodules when cytology continued to be benign (1-3,7). To our knowledge, this is the first study evaluating the frequency of malignancy specifically in the case of nodules with highly suspicious US features, but with two FNA showing benign cytology.

The frequency of malignancy was 2% in the present series. A histological diagnosis could not be obtained in 86 nodules. Although we cannot ensure the absence of malignancy in these cases, considering that all nodules had three benign cytologies, we believe that a malignant nature is very unlikely (5,6,8). Furthermore, the nodules measured up to 4 cm and none of the patients had a family history of thyroid carcinoma, was exposed to radiation during childhood or adolescence, had hypercalcitoninemia or a nodule detected incidentally by FDG-PET for the staging of oncological disease. Additionally, the samples were analyzed by pathologists experienced in thyroid cytology. We therefore do not believe that the rate of malignancy is overestimated. In contrast, the rate of false-negative cytology may even be higher in patients at high clinical risk for thyroid malignancy or in the case of large nodules.

The rate of malignancy is approximately 15-20% when the first FNA reveals benign cytology, but the nodule is highly suspicious on US (1-4), findings justifying a second FNA (1-4). On FNA repetition, cytology continued to be benign in approximately 80% of the patients included in the present study. The third FNA, although it permitted the diagnosis of malignancy in 2% of the patients, only confirmed the previous results in 90% of the patients and led or contributed to unnecessary surgery in 8% (non-benign cytology, but histology without malignancy). Thus, a third FNA would not be interesting. On the other hand, in view of the non-negligible risk of malignancy, in contrast to the ATA recommendation [“If a nodule has undergone repeat US-guided FNA with a second benign cytology result, ultrasound surveillance for this nodule for continued risk of malignancy is no longer indicated” (4)], we believe that the continuation of follow-up consisting of US at intervals of 2 years may still be adequate in these cases. In addition to the risk of malignancy, papillary carcinomas with highly suspicious US appearance seem to have a poor prognosis (9).
